# Exploring neurogastronomy: an analysis using the word association test and free word association

**DOI:** 10.3389/fpsyg.2026.1771119

**Published:** 2026-02-24

**Authors:** Ceyhun Uçuk, Özkan Süzer, Charles Spence

**Affiliations:** 1 Şarköy Vocational School, Namık Kemal University, Tekirdağ, Türkiye; 2 Gastronomy and Culinary Arts Department, Gaziantep University, Gaziantep, Türkiye; 3 Gastronomy and Culinary Arts Department, Karabük University, Karabük, Türkiye; 4 Department of Experimental Psychology, University of Oxford, Oxford, United Kingdom

**Keywords:** gastronomic neuroscience, gastronomy, gastrophysics, neurogastronomy, neuroscience

## Abstract

**Introduction:**

This study expands our understanding of neurogastronomy’s various dimensions and addresses a critical gap in the literature concerning its conceptual structure. However, as a field of research, neurogastronomy lacks conceptual clarity despite its growing popularity. Drawing on Semantic Network Theory, according to which cognitive associations define conceptual understanding, this study investigates the field’s cognitive structure.

**Methods:**

Word Association Tests were conducted with 327 gastronomy students in Turkey.

**Results:**

The analysis yielded 16 codes and 6 themes, with “sensory experience and taste perception” emerging as dominant. The findings demonstrate that neurogastronomy functions as a fragmented rather than a cohesive concept within the cognitive network.

**Discussion:**

By empirically mapping the field’s conceptual boundaries, this study provides a foundation for clarifying terminology and informing future theoretical and applied research.

## Introduction

Two decades ago, in 2006, Gordon Shepherd coined the term ‘neurogastronomy’, drawing attention to the fact that the perception of flavour involves complex neural processes. He further highlighted how these processes are deeply intertwined with the experience of flavour, showcasing the multifaceted nature of flavour perception. In the research outined here, the concept of ‘neurogastronomy’ (Shepherd, 2012) and the framework of multisensory perception (Spence, 2017) are used to comprehend the complex nature of taste and flavour perception. Flavour is not merely an experience detected by taste receptors on the tongue and elsewhere within the oral cavity (Spence, 2022), but is instead a multifaceted multisensory process involving the interaction of diverse elements within the sensory system. The theoretical framework of the study elucidates the multisensory integration taking place in the brain and the gastronomic experiences that are formed as a result. Neurogastronomy posits that taste and flavour perception are influenced by both biological and cognitive brain processes (Shepherd, 2012). The experience of flavour is influenced not just by the taste buds but also by olfactory, tactile, cognitive, visual, and auditory elements, and is shaped by memory, anticipation, and cultural influences (Spence, 2017). Furthermore, the perception of taste and flavour is also influenced by biological variables, such as the intestinal microbiota and the hormone system (Koskinen et al., 2018). In a study conducted by Morrot et al. (2001), changing the colour of white wine to resemble a red wine was shown to influence which terms participants (oenology students) chose to describe its taste and flavour. Wang and Spence (2019) demonstrated that giving a white wine a rose-pink tint similarly influenced multisensory flavour perception. Furthermore, research has demonstrated the impact of auditory cues (e.g., cracking sound) on freshness perception as well as on the flavour of food (Spence et al., 2019; Zampini and Spence, 2004). The present study investigates Turkish gastronomy students’ perceptions of neurogastronomy and the assessment of multisensory perception systems through the Word Association Test (WAT), which allows for an in-depth analysis of cognitive associations and conceptual understandings of sensory integration, flavour perception, and the principles of neurogastronomy.

The present study is grounded in Semantic Network Theory (Collins and Loftus, 1975; Dong and Qi, 2012), according to which, concepts in human cognition are represented as nodes within a mental network, where the strength of associations between these nodes determines the speed and likelihood of neural activation. According to this theory, when an individual encounters a stimulus-such as the term ‘neurogastronomy’, related concepts that are strongly linked in the semantic network become more readily accessible, whereas weaker or less frequently activated concepts may remain peripheral. This theoretical framework provides a structured approach to analyzing the cognitive representation of neurogastronomy among gastronomy students by examining the words they choose to associate with the concept. By using a Word Association Test (WAT), we investigate the hierarchical organization of neurogastronomy-related concepts in the participants’ mental lexicon, revealing dominant associations (e.g., flavour, taste, perception) and less prominent but theoretically significant dimensions (e.g., health, creativity). The findings contribute to understanding how neurogastronomy is conceptualized and which dimensions are more strongly integrated into students’ cognitive networks, thereby offering insights into educational strategies and interdisciplinary connections within gastronomy research.

Among all the factors influencing the preference and evaluation of a dish, the concept of flavour stands out the most (Spence, 2017). Note that mostly when people (and that includes scientists) talk about taste, what they actually mean is flavour (Spence et al., 2015). The question of why people like different tastes/flavours, has long fascinated researchers. According to Shepherd (2012), flavour perception results from the combination and interpretation of olfactory and gustatory stimuli in the brain. However, this alone is not sufficient to explain the multisensory experience of flavour (Spence, 2017, 2024). For instance, piperine, the primary trigeminal stimulant in black pepper, has been shown to influence the perception of saltiness (Moss et al., 2023). Shepherd’s effort to define flavour exclusively through electrical activity in the brain^1^ invites critical scrutiny regarding the adequacy of such an approach.

When soluble substances in the mouth activate the taste receptor cells that are found in the taste buds on the tongue as well as elsewhere in the oral cavity (Spence, 2022), individuals experience a sensation through the activation of the gustatory system (ISO, 2008). Within this framework, previous research in neurogastronomy has examined taste perception and flavour formation from a neuroscientific perspective, employing methods that focus on underlying brain mechanisms (Campinho et al., 2023; Pribic and Azpiroz, 2018; Spence and Piqueras-Fiszman, 2014).

Within neurogastronomy research on the perception of flavour, subjective (sensory analysis, qualitative assessment, etc.) and objective measures (electroencephalography, facial expression analysis, eye-tracking devices, etc.) can, and have, been used both separately and together (Uçuk, 2022; Ucuk et al., 2025; Piqueras-Fiszman et al., 2013). For example, in a study on the relationship between sound and taste, auditory stimuli, such as crunching or sizzling sounds, have been shown to affect individuals’ perception of food as well as enhancing their enjoyment (Spence et al., 2019, p. 282).

Neurogastronomy has undoubtedly gained popularity as scientific field in recent years. The enrichment of sensory perceptions and experiences significantly enhance the quality of gastronomic experiences, and the acceleration of research in neurogastronomy offers notable potential for innovative applications and advanced discoveries for future research (Muskat et al., 2024). However, it is important to acknowledge the objection that neurogastronomy can be seen as just another instance of the “neuromania,” a term used by some scholars to warn against overextending neuroscience into areas that are not empirically supported (Legrenzi and Umiltà, 2011). Addressing these critiques while maintaining a focus on interdisciplinary rigor will be crucial for the field’s continued development and credibility.

In order to understand the academic visibility of the concepts thought to be most associated with neurogastronomy over the years, the frequency of appearance of 10 prominent words in this field in Google Scholar was examined. In this analysis, which covers publications between 1960 and 2024, the academic distribution of the determined terms in certain time periods was investigated. The obtained data reveal the change in concepts related to neurogastronomy in the academic literature over time, and this distribution is presented in Table 1. The data presented in Table 1 highlights a general rise in the use of neurogastronomy-related terms over recent years, reflecting the field’s growing popularity and progression. The frequently used terms in this table illustrate emphasizing the interdisciplinary nature of neurogastronomy. The analysis of the scientific literature reveals a notable increase in the usage of the term neurogastronomy and its associated concepts from 1960 to 2024 (see Table 1). The findings indicate a growing academic interest in neurogastronomy over time, reflecting its expanding role in interdisciplinary research. The increasing frequency of these terms suggests a heightened recognition of neurogastronomy in studies related to consumer behaviour, sensory perception, and food science.

**Table 1:** Frequency of occurrence the term neurogastronomy and related terms in scientific literature (1960–2024).

Words	1960–1970	1971–1980	1981–1990	1991–2000	2001–2010	2011–2024
Neurogastronomy	0	0	0	0	3	817
Flavour	181 k	232 k	49 k	143 k	299 k	58 k
Food	481 k	564 k	514 k	938 k	571 k	851 k
Taste	35 k	92 k	211 k	430 k	1.480 k	2.100 k
Perception	163 k	443 k	647 k	1.250 k	1.610 k	2.170 k
Brain	307 k	615 k	509 k	616 k	677 k	1.300 k
Sense	311 k	357 k	569 k	1.150 k	1.430 k	2.270 k
Smell	17 k	233 k	388 k	113 k	799 k	144 k
Feeling	89 k	160 k	172 k	615 k	1.310 k	2.340 k
Memory	148 k	476 k	673 k	1.160 k	1.250 k	2.070 k
Science	503 k	298 k	239 k	362 k	472 k	778 k

This table is based on publication data retrieved from Google Scholar on 4 January 2025. Citation: Google Scholar. (2025). Search results for selected keywords. Retrieved January 4, 2025, from Google Scholar database.

While studies on neurogastronomy generally progress in the form of experimental research, how this concept is perceived is unknown. When knowledge of neurogastronomy was examined recently amongst five-star hotel chefs, it was found that most of them were generally unaware of the concept (Unal, 2024). While neurogastronomy has predominantly been examined through experimental and neuroscience methods, the ways in which this concept is socially constructed, culturally interpreted, and educationally internalized remain underexplored. Food perception and flavour are not only biological phenomena but are also shaped by cultural learning, professional training, and shared gastronomic norms, as extensively discussed in anthropological and sociocultural studies of food and taste (e.g., Fischler, 1988; Spence and Youssef, 2018; Sutton, 2010). From this perspective, neurogastronomy should not be regarded solely as a biomedical framework, but also as a concept whose meaning and relevance are negotiated within specific social and educational contexts.

Gastronomy students represent a particularly relevant group for examining these dynamics, as they are situated at the intersection of scientific knowledge, culinary practice, and cultural transmission. During their education, students are exposed to emerging scientific concepts-such as neurogastronomy-while simultaneously internalizing professional culinary norms and sensory vocabularies. Investigating how gastronomy students cognitively conceptualize neurogastronomy therefore provides insight not only into individual perception, but also into how interdisciplinary knowledge is translated into gastronomic education and professional identity formation. Despite the growing body of experimental research in neurogastronomy, there is a notable lack of studies examining how the concept itself is perceived, structured, and hierarchically organized in the minds of individuals engaged in gastronomic training. Addressing this gap in the literature, the present study adopts a cognitive-semantic approach to explore gastronomy students’ mental representations of neurogastronomy, thereby integrating neuroscientific, educational, and sociocultural perspectives within a unified analytical framework.

Moreover, as yet, we are aware of no research that has examined the dimensions through which the notion of neurogastronomy is understood, or the particular cognitive associations and meaning structures that are most salient to individuals. The aim of the present research was therefore to reveal the dimensions through which the concept of neurogastronomy is perceived by individuals related to the field of gastronomy and the hierarchy among these dimensions. In this way, the dimensions through which the concept of neurogastronomy is understood will be revealed, and an important gap in the literature filled. In this context, usable data were collected from 327 gastronomy students from 26 different universities operating in Türkiye using WAT and FWA. The students were asked to write down 10 words they associate with the concept of neurogastronomy, and the responses were analyzed using the MAXQDA program. Then, they were categorized and combined under codes and themes.

Modern gastronomy is evolving from traditional approaches, reflecting a continual process of change in the field. While studies of neurogastronomy are concerned with understanding how the brain interprets and processes sensory stimuli, molecular gastronomy is concerned with the scientific transformation of food through creative culinary methods (Spence and Youssef, 2018; Velasco et al., 2021). Accordingly, neurogastronomy has emerged at the intersection of gastronomy and cognitive neuroscience, integrating insights from both domains to investigate sensory and cognitive influences on food experiences (Herz, 2016; Shepherd, 2006; Spence, 2012; Uçuk, 2022; Ucuk et al., 2025).

Neuroscience techniques are also used in studies investigating the effects on preference of presenting the same dish in forms prepared with different designs. For example, in the studies conducted by Berčík et al. (2021) and Spence (2017), neuroscience techniques have been used to investigate the effects of food presented in forms prepared with different designs on people’s preferences and emotions. The neuroscience method is beneficial as it offers objective insights into the fundamental principles of multisensory perception and flavour development. By incorporating neuroscience, the research transcends subjective evaluations, facilitating a more profound comprehension of how multisensory interactions influence culinary experiences. In the study conducted by Uçuk (2022), the effects of food presented in different formal forms on people’s perception of liking were also investigated. In the latter study, electroencephalography and facial expression analysis techniques from neuroimaging methods were used simultaneously. The research findings demonstrated that presenting the same dish in different forms resulted in differences in people’s perception of taste.

The role of sensory experiences, especially basic senses such as taste and smell, in gastronomic experiences is also related to the concept of crossmodal perception (Ai and Han, 2022). For example, in Spence’s (2019b) study, the relationship between colour and taste was examined, and significant findings were presented on how the interaction of these two senses affects flavour perception. Spence (2019b) emphasizes that colours can alter taste perception and that this can have significant effects on consumer behaviour. Additionally, research in the field of neurogastronomy provides important insights into how sensory signals that affect consumers’ taste preferences come together (Lahne, 2013; Uçuk, 2022; Ucuk et al., 2025).

Research on sensory training reveals actionable insights into enhancing sensory perceptions, which are foundational to neurogastronomy. For example, Spence (2021) reviewed the literature showing that targeted sensory training programs, such as aroma identification exercises, significantly improve individuals’ ability to discern complex flavour profiles. These improvements not only enrich gastronomic experiences but also enhance the appreciation of fine dining. Additionally, neuroscientific tools, such as electroencephalography (EEG) and functional magnetic resonance imaging (fMRI) (Lorentzen et al., 2021), have been used to analyze consumer responses to food advertisements, enabling the design of marketing strategies tailored to emotional triggers (Spence, 2015). Nonetheless, the used of these measurement instruments faces criticism for removing the dining experience from its customary context. Spence (2015) asserts that comprehending a complicated phenomenon like taste through neuroimaging techniques is exceedingly challenging due to the inherently social nature of human eating behaviour.

Despite these advances, the rapidly expanding literature in neurogastronomy has yet to achieve conceptual coherence. While empirical studies have successfully demonstrated how sensory training and neuroscientific methods can enhance gastronomic experiences, considerably less attention has been paid to how the concept of neurogastronomy itself is understood and structured. In particular, there is a lack of empirical research examining the cognitive dimensions through which neurogastronomy is perceived and which aspects of the concept are most salient to individuals. Addressing this gap, the present study investigates the cognitive structure of neurogastronomy using Semantic Network Theory and Word Association Tests, thereby providing a systematic framework for clarifying its conceptual boundaries.

## Materials and methods

The present study was designed to explore people’s associations with the term ‘neurogastronomy’, as this has not been adequately researched previously. In order to assess the perception of neurogastronomy among industry experts and gastronomy students, the Word Association Test (WAT) and Free Word Association (FWA) methodologies were used. The WAT is a technique that is commonly-used to help uncover people’s perceptions and beliefs regarding specific ideas or concepts (Ares et al., 2008; Hovardas and Korfiatis, 2006). FWA emphasises people’s more spontaneous and unrestrained reactions to a specific word (Ross, 2003). The combined application of these two methods enhanced our understanding of the range of associations that people (in particular, Turkish gastronomy students) hold related to the idea of neurogastronomy. The use of WAT and FWA aligns with Semantic Network Theory (Collins and Loftus, 1975; Segev, 2022; Yao et al., 2022), according to which concepts are represented as interconnected nodes within cognitive structures, with stronger associations being more readily activated. By using these techniques, the present study investigates how the concept ‘neurogastronomy’ is structured in the mental lexicon of participants, revealing the hierarchical relationships and activation strengths between various different concepts. The results contribute to furthering our understanding concerning how individuals cognitively organize and retrieve information related to neurogastronomy within their semantic networks.

### Participants

The sample group was comprised of those associated with the field of gastronomy, given that the term ‘neurogastronomy’ remains is unfamilar in society at large. In particular, the study focused exclusively on students pursuing academic education in food and gastronomy. A minimum of 300 valid responses is required to examine in order to produce significant conceptual maps in word association tests (Memon et al., 2020). To maintain a significant sample size, we referenced similar research in the literature (Çetin and Bora, 2022; Onat and Keskin, 2023). We collected data from 736 individuals across 26 colleges to minimise attrition.

The decision to focus on gastronomy students was both theoretically and methodologically motivated. As neurogastronomy is an emerging interdisciplinary concept located at the intersection of neuroscience and gastronomy, students receiving formal education in this field are uniquely positioned to engage with both its scientific foundations and its culinary applications. Examining this group therefore enables an assessment of how such interdisciplinary concepts are cognitively internalized during professional training.

That having being said, it should also be acknowledged that limiting the sample to gastronomy students may introduce a degree of disciplinary specificity, which may restrict the generalizability of the findings to wider populations. However, given the exploratory nature of the study and the relatively low level of societal familiarity with the concept of neurogastronomy, this population was considered appropriate for an initial conceptual mapping. Accordingly, this focus is treated as a deliberate methodological choice rather than a methodological limitation, while implications for generalizability are addressed in the Discussion section.

### Data collection and analysis procedure

**Figure 1:**
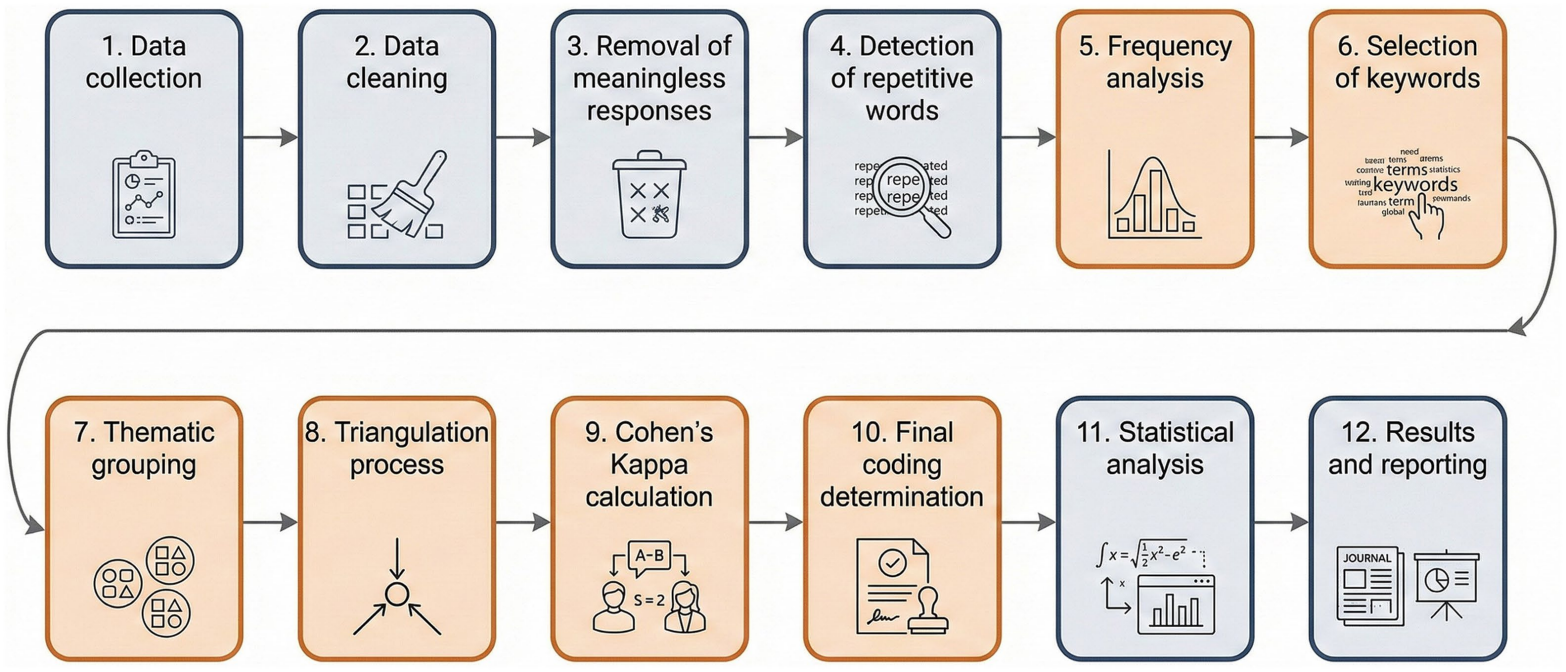
Data collection and analysis process.

Data were gathered electronically using Qualtrics. The analytical procedure followed a structured, multi-stage process (see Figure 1). The participants were allotted 10 s in which to deliver each response. This duration was derived from the optimal time (10 s) used in analogous association analyses conducted previously (Ares et al., 2015). The participants were instructed to answer the question “What does neurogastronomy evoke for you?” using 10 distinct terms. Responses were analysed via Maxqda software, frequency analysis was conducted, and data were categorised into specific primary themes (see Figure 1). To avoid priming effects and ensure that associations reflected participants’ existing cognitive representations, no definition or explanatory information regarding the concept of neurogastronomy was provided prior to data collection. Participants were not assessed for prior formal knowledge of the term, as the objective of the Word Association Test was to capture spontaneous and self-generated associations, irrespective of participants’ level of familiarity. This approach is consistent with previous WAT and FWA studies aiming to reveal latent semantic structures rather than informed or instructed responses.

The analysing procedure followed a structured approach consisting of several key steps. Initially, data cleaning was conducted in order to eliminate nonsensical responses or non-lexical items. Next, a frequency analysis was performed in order to identify the most commonly repeated words, specifically focusing on those that appeared at least five times. Subsequently, keywords were categorized into principal themes based on the existing literature. During the theme creation phase, only those words with a repetition frequency of five or more (a total of 110 words) were included in the analysis, while those with lower repetition counts (404 words) were excluded. This methodological approach aligns with previous studies that have used similar criteria for thematic categorization (Andrade et al., 2016; Ares et al., 2015).

Due to the diversity of the data, a triangulation process was implemented, and thematic analyses were conducted to ensure the robustness of the findings (Apostolidis, 2003; Guerrero et al., 2010). Initially, the researchers performed a semantic analysis of the words based on their individual interpretations, developing distinct categories independently. These categories were then compared, and the final classification was established by means of consensus. Ultimately, the researchers consolidated the categories under overarching themes, aligning with established methodological approaches (Andrade et al., 2016). To further validate the reliability of the categorization process, inter-coder agreement was assessed using Cohen’s Kappa coefficient, which yielded a value of 0.78, indicating a substantial level of consistency, reinforcing the robustness of the thematic analysis.

## Results

The study participants were generally young, with the majority of participants in the 18–22 years of age range, due to the sample being recruited from amongst university students (see Table 2 for demographic data).

**Table 2:** Demographic characteristics of the participants.

Demographics	Groups	*n*	%	Demographics	Groups	*n*	%
Age (years)	18–22	232	71	Gender	Female	232	71
	23–27	53	16		Male	95	29
	28–32	17	5	Profession	Student	327	100
	32+	25	7				

The findings obtained from the word analysis are reported in two stages. First, the percentages of words frequently repeated by the participants are shared (see Table 3 for the word frequencies based on participants’ responses) (note that words with a frequency of 4 or less (a total of 404 words) are not included).

**Table 3:** Word frequency counts and percentages.

Word	Frequency	%	Word	Frequency	%
Flavour	231	6.92	experience, texture	24	1.44
Food	227	6.80	connotation	23	0.69
Taste	196	5.87	synesthesia	21	0.63
Perception	176	5.27	analysis, border	20	1.20
Brain	163	4.88	feel, visual	19	1.14
Sense	136	4.07	cognition, colour, effect	17	1.53
Smell	117	3.51	rapport	16	0.48
Feeling	102	3.06	behaviour, eating	15	0.90
Memory	91	2.73	chemical, drink, organ	14	1.26
Science	69	2.07	consciousness, intuition, relationships, seeing, tasting	13	1.95
Mind	55	1.65	habit, marketing, molecular, palate, stomach	12	1.80
Senses	48	1.46	cognitive, feelings, happiness	11	0.99
Pleasure	46	1.40	aroma, research, satisfaction, thought	10	1.20
Kitchen	36	1.10	detail, difference, fusion, health, heat, influence, intelligence, mental, neuron, combination	9	2.70
Art, sensory	31	1.90	chef, cooking, hunger, innovation, scientific, technology	8	1.44
Presentation	29	0.87	auditory, drinking, examination, future, illness, language, neuroscience, system, thinking	7	1.89
Image	27	0.81	appetite, cell, cerebral, culture, different, experiment, love, person, plate, psychology, subconscious, sweet	6	2.16
Gastronomy	25	0.75	appeal, biology, creativity, development, do, sight, gourmet, interaction, message, neurology, quality, request, satiety, sensation, sound, touch, vote, warning	5	2.70

Note that words with the same number of repetitions (e.g., experience, tissue) is that in same box.

The term with the highest frequency in Table 3 is ‘flavour,’ which was mentioned 231 times. Considering that understanding how flavour is represented/computed in the brain is one of the main aims of neurogastronomy, this result makes perfect sense. This term is followed in order by ‘food’, ‘taste’, and ‘perception’. In other words, the words most frequently repeated by the participants are commonly-used terms related to the field of gastronomy. Similarly, as shown in Table 3, the words with the lowest frequency (5) were ‘appeal’, ‘biology’, ‘creativity’, ‘development’, etc. This indicates that the participants in the present study associated the concept of neurogastronomy with various ideas, reflecting both quantitative distinctions and terminological diversity. The fact that neurogastronomy is still a relatively new term/field of study is likely a key contributor to the variation that exists in this field. In addition to the dominance of core sensory-related terms, the low frequency of words such as biology, creativity, marketing, and health indicates that participants’ associations tend to cluster around immediate perceptual and experiential aspects rather than systemic, applied, or translational dimensions of neurogastronomy. This imbalance suggests that the concept is currently understood in a relatively narrow sensory-cognitive frame, with limited integration of its applied and interdisciplinary potential.

**Figure 2:**
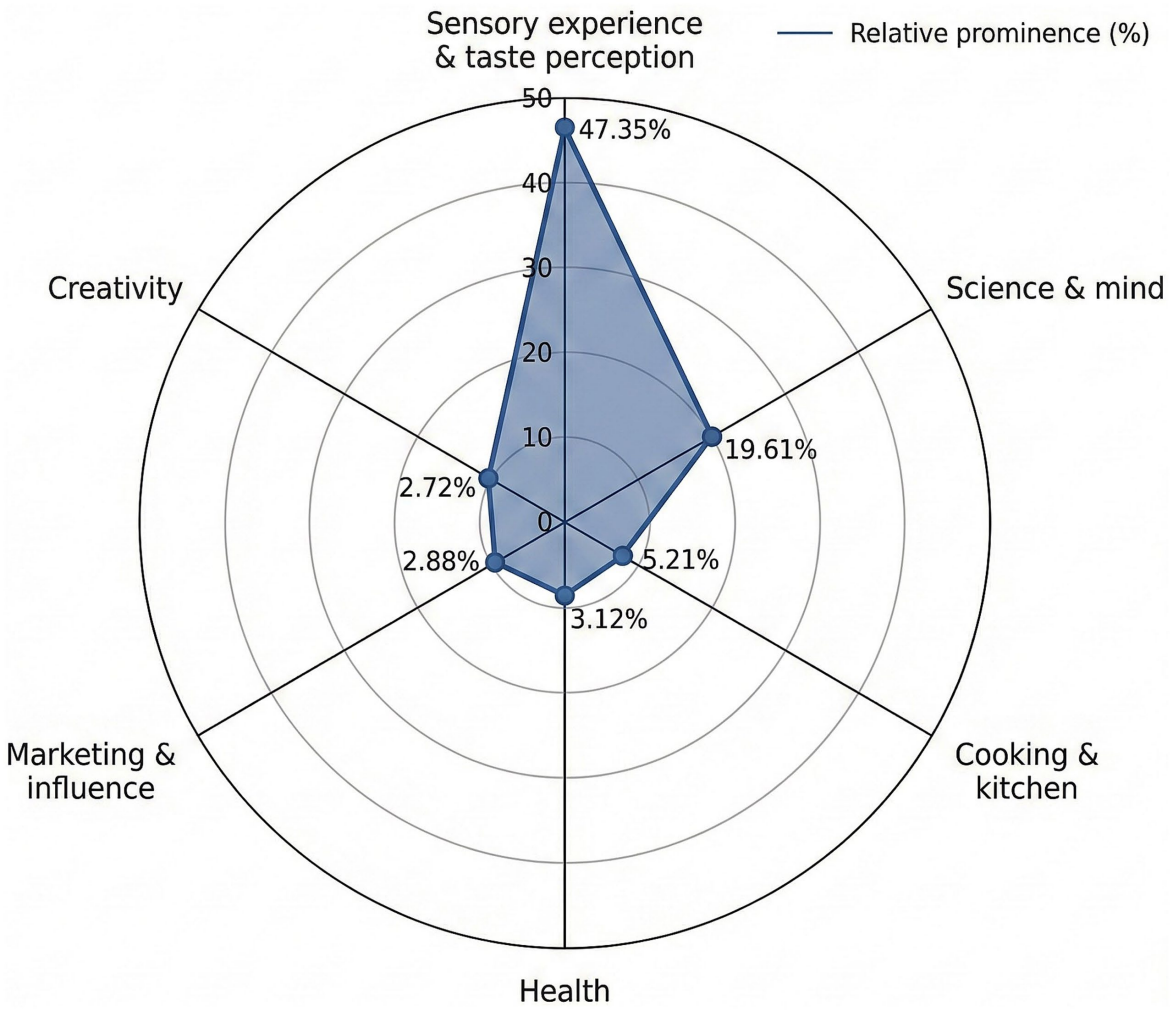
Relative prominence of neurogastronomy dimensions among gastronomy students [this radar (spider) chart illustrates the proportional distribution of six main neurogastronomy dimensions derived from the WAT and Free Word Association results. The figure highlights the dominance of sensory experience and taste perception, while dimensions such as health, marketing and influence, and creativity appear comparatively peripheral].

Table 4 presents a detailed categorization of dimensions, including sensory experience, scientific concepts, culinary aspects, health, marketing, and creativity, with associated word frequencies and percentage distributions, offering insights into their relative significance to the participants. After analysis, 16 categories were eventually identified, and these were grouped under 6 dimensions, amongst which ‘sensory experience and taste perception’ and ‘science and mind’ stand out (see Figure 2).

**Table 4:** Categorization of linked words into dimensions and categories with their frequencies and percentages.

Dimension	Categories (words)	Frequency	%
Sensory experience and taste perception	Taste dynamics (flavour, taste, smell, palate, aroma, sweet, tasting)	585	17.53
	Sensory perception (perception, sensation, feeling, senses, sensory, synesthesia, sense, feel)	538	16.16
	Eating experience (food, fusion, appetite, satiety, pleasure, hunger, image, experience)	352	10.57
	Visual perception (visual, colour, seeing, sight, connotation)	77	2.31
	Sensory details (detail, touch, auditory, sound)	26	0.78
Total		1,578	47.35
Science and mind	Neuroscience (brain, neuron, molecular, chemical, neuroscience, system, cerebral, neurology, cell)	229	6.86
	Cognitive processes (consciousness, cognition, cognitive, thought, intelligence, memory, thinking, mind, mental, subconscious)	228	6.84
	Scientific research (science, research, scientific, technology, examination, analysis, experiment, future)	135	4.05
	Behaviour and psychology (behaviour, psychology, effect, intuition, feelings)	62	1.86
Total		654	19.61
Cooking and kitchen	Culinary arts (kitchen, chef, cooking, gourmet, gastronomy, plate, presentation, heat)	126	3.80
	Food culture (eating, drink, drinking, culture, quality)	47	1.41
		173	5.21
Health	Health (health, illness, satisfaction, happiness, habit, stomach)	61	1.83
	Biology (biology, tissue, organ)	43	1.29
Total		104	3,12
Marketing and influence	Marketing (marketing, influence, appeal, development, vote, border)	56	1.68
	Communication and interaction (message, language, interaction, request, warning, relationship)	40	1.20
Total		96	2.88
Creativity	Creativity (art, creativity, difference, love, person, combination, innovation, rapport)	90	2.72
Total		90	2.72

Note that categories consist of words that were linked to each other while dimensions were made up of categories that were linked to each other.

Beyond the prominence of dominant dimensions, the results are equally informative in terms of what remains conceptually marginal or weakly represented. Notably, dimensions related to health (3.12%), creativity (2.72%), and marketing and influence (2.88%) appear at the periphery of participants’ cognitive networks. Given the growing emphasis on personalized nutrition, wellbeing, and experiential innovation in contemporary neurogastronomy research, the relatively low salience of these dimensions suggests a conceptual gap rather than a lack of relevance. From a semantic network perspective, such peripheral positioning indicates weaker associative links and lower activation strength within the participants’ mental lexicon. This pattern implies that although Turkish gastronomy students strongly associate neurogastronomy with sensory experience and neuroscientific processes, they have not yet fully integrated its broader implications for health, creativity, and consumer engagement into a cohesive conceptual structure. These absences are analytically significant, as they point to areas where neurogastronomy is under-conceptualized rather than absent from the field itself.

The most prominent theme is sensory experience and taste perception, consisting of five categories (taste dynamics, sensory perception, eating experience, visual perception, and sensory details). When evaluated on a percentage basis, the dimensions of taste dynamics and sensory perception stand out, suggesting that this is what Turkish gastronomy students consider key when thinking about the meaning of the term neurogastronomy. The distribution of dimensions and categories is further visualized in Supplementary Figure S1, which highlights the dominance of sensory-related associations and the peripheral role of health and creativity.

The second prominent theme is that of ‘science and mind’. The categories and words included in this theme are generally targeted at understanding the scientific and mental processes that underlie flavour perception. Under this theme, there are four categories under this theme (neuroscience, cognitive processes, scientific research, and behaviour and psychology) and neuroscience (6.86%) and cognitive processes (6.84%) contribute statistically more than scientific research (4.05%), behaviour and psychology (1.86%). These observations highlights the connection between food perception and neurogastronomy amongst Turkish gastronomy students.

The third theme, ‘cooking and kitchen,’ encompasses the categories of culinary arts and food culture. When this theme and its subcategories are examined, the practical and cultural aspects of cooking come to the forefront. From this, it can be understood that when neurogastronomy is mentioned, cooking practices and culture are also amongst the categories that come to mind amongst Turkish gastronomy students.

The fourth theme relates to ‘health’, formed in order of the health and biology categories. This theme indicates that Turkish gastronomy students associate neurogastronomy with health. The fifth theme is ‘marketing and influence’, consisting of the categories of marketing, communication, and interaction. This theme highlights the role of neurogastronomy in understanding consumer behaviour and perceptions while also emphasizing its potential applications in food marketing. The findings of the present study (e.g., marketing and ınfluence dimension) are consistent with this perspective. The sixth theme identified within the scope of the present study was ‘creativity’. Neurogastronomy examines how food affects sensory perception and brain processes, while the creativity theme emphasizes innovation and unique experiences. The elements of art and innovation contribute to a deeper understanding of both the scientific and artistic dimensions of neurogastronomy by fostering a sense of connection between individuals. The findings suggest that Turkish gastronomy students concentrate on specific aspects of neurogastronomy, such as sensory perception and scientific procedures, while possibly neglecting others, such as health and creativity. (With regard to this specific situation, this research underscores the necessity for more extensive education and interdisciplinary inquiry to fully realize the potential of neurogastronomy as a holistic domain in Turkey.)

**Figure 3:**
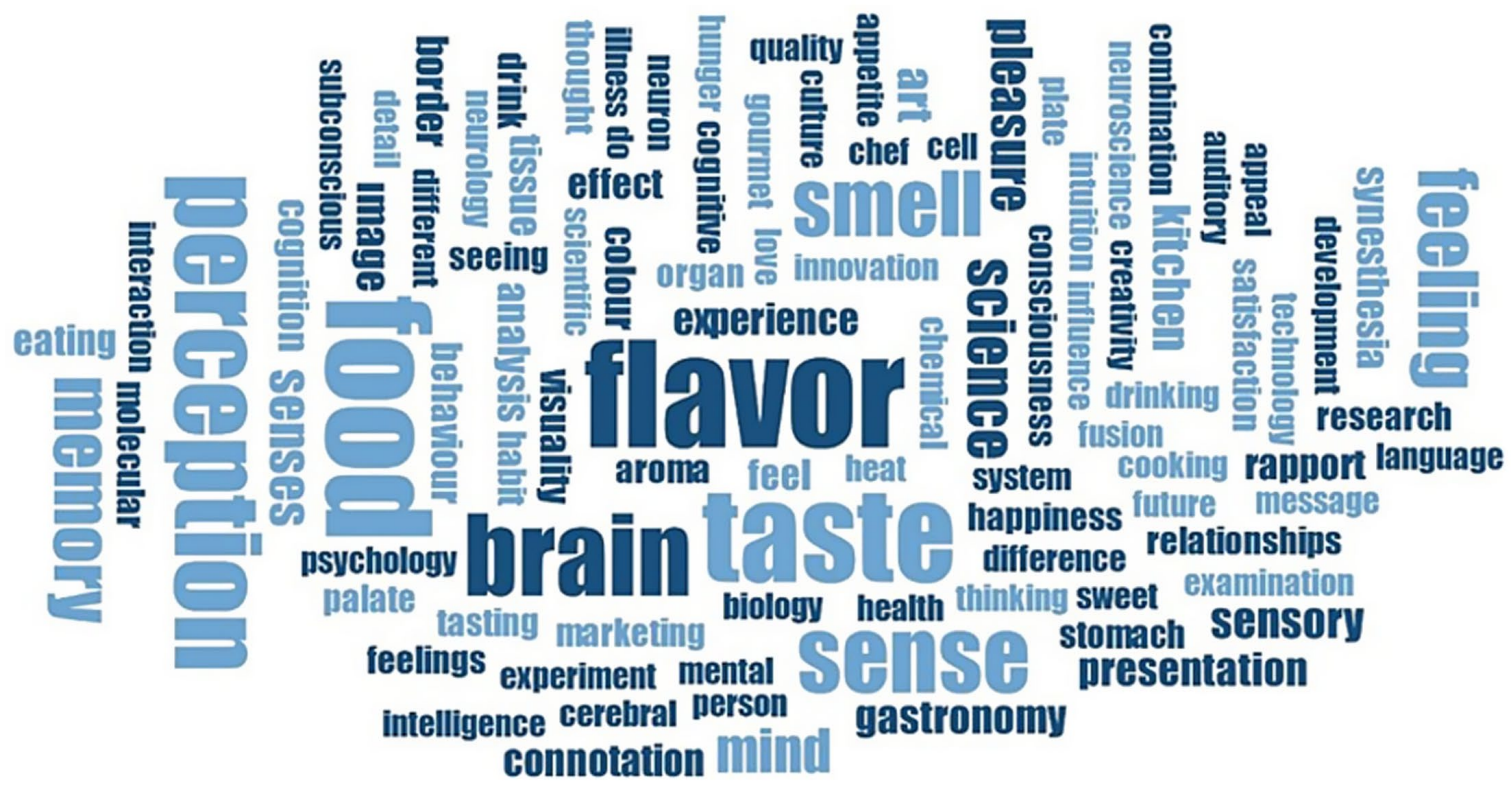
Word cloud representation of the words expressed related to neurogastronomy. In the figure, the words with the highest frequency are seen in larger typeface. Notice how words like flavour, perception, taste, and food, which are repeated most frequently, stand out in the word cloud.

After determining the word frequencies and rankings, the word cloud method was used to visualize the data. All of the words included in the research were subjected to analysis. Below, in Figure 3, shows the word cloud generated by the Maxqda software based on the frequency of some of the words given by the participants in their responses.

## Discussion

The findings of the present study indicate that neurogastronomy is most frequently associated with the words ‘flavour’, ‘taste’, ‘perception’, ‘brain’, and ‘science’ by the participants (constituting a wide selection of Turkish gastronomy students). According to Shepherd (2006), flavour is perceived and shaped by the brain through complex neuroscientific processes. Though researchers have criticized the suggestion from Shepherd and other that ‘flavour is in the brain’ (e.g., Small, 2012; Smith et al., 2017), the ‘science and mind’ theme obtained in the present study demonstrates the strong connection between neurogastronomy and neuroscience, and that the Turkish gastronomy students who were participated are clearly aware of this connection. Fooladi et al. (2019) investigated the dynamics of collaboration between chefs and researchers regarding access to and utilisation of scientific knowledge, demonstrating that culinary professionals use a knowledge-driven and innovative methodology in their engagement with science. Nonetheless, it was underscored that time management, resource scarcity, and challenges in interdisciplinary communication constrain these collaborations. In the present study, the awareness amongst Turkish gastronomy students of the relationship between neurogastronomy and neuroscience serves as a significant indicator of the academic perception of the topic. This scenario offers a significant framework for comprehending the adoption of scientific terminology in gastronomy education and its correlation with the cognitive processes of culinary arts students.

While taste and flavour are predominantly discussed within neuroscientific frameworks in neurogastronomy, anthropological perspectives highlight that taste is also a culturally learned and socially negotiated phenomenon. From an anthropological standpoint, taste is a form of embodied knowledge shaped through cultural practices, socialization, and shared culinary norms (Fischler, 1988; Sutton, 2010). The strong association of neurogastronomy with sensory enjoyment and perception observed among Turkish gastronomy students may reflect culturally embedded understandings of what constitutes “good food” and professional culinary competence. This perspective suggests that students’ emphasis on flavour and sensory pleasure aligns with the anthropological view of taste as a marker of identity, expertise, and cultural belonging. Integrating such insights extends the interpretation of the findings beyond a purely neuroscientific account and supports a more genuinely interdisciplinary understanding of neurogastronomy, where biological mechanisms, cultural meanings, and professional training intersect.

Health, a significant element within the study of neurogastronomy, was infrequently mentioned by the Turkish gastronomy students who took part in this study. This result suggest that the Turkish gastronomy students either fail to acknowledge its significance or do not devote sufficient attention to it currently. Neurogastronomy is also related to the topic of neuromarketing; however, it turned out that the marketing theme was a relatively low-frequency response amongst the respondents who were quizzed in the present study. The marketing dimension of neurogastronomy ranks lower than most other dimensions among the participants assessed in the present study, suggesting that neurogastronomy is not perceived to be synonymous with consumer neuroscience (Spence, 2020), by Turkish gastronomy students.

The findings of the neurogastronomy-focused analysis indicate that the Turkish gastronomy students evaluated in this study predominantly used expressions emphasising sensory enjoyment in food, while referencing health considerations to a lesser extent. Achieving a balance between the dimensions of health and sensory pleasure is crucial for the development of the concept of neurogastronomy. Previous research, such those by Roininen et al. (2006) and Guerrero et al. (2010) underscore the interrelated and supplementary roles of health factors and sensory attractiveness in shaping food preferences and improving overall wellbeing. The findings also show that sensory experience and vision, was commonly linked to neurogastronomy by the survey respondents, in line with work of Delwiche (2003) with sensory professionals, where the importance of vision was also apparent. An analysis of the dimensions reveals that creativity is the least mentioned theme among Turkish gastronomy students. The fact that creativity is not immediately associated with neurogastronomy is a point that requires further attention. This is particularly important as the concept of creativity in food is becoming increasingly significant. While high-quality food was traditionally perceived as expensive, it is now increasingly defined by its creativity and uniqueness (Otero, 2018).

Although beyond the scope of the present research, it is suggested that oral, nasal, or gut microbiota may also play a role in flavour perception (Koskinen et al., 2018; Spence, 2019a,b; Taylor et al., 2020). This indicates that neurogastronomy alone may be insufficient to fully explain the complexities of taste and flavour perception. This highlights the need for an interdisciplinary approach, as taste is seen as a neural process in natural sciences, a cultural construct in social sciences, and a fusion of sensory and cognitive processes in philosophy (Hedegaard, 2019). The Word Association Test (WAT) results further support this perspective, as high-frequency terms such as “memory” and “pleasure” suggest that flavour perception extends beyond neural mechanisms to include sensory, cognitive, and emotional factors. Additionally, the presence of words like “chemical” and “organ” highlights potential microbiological influences, reinforcing the idea that a more integrated framework is necessary to understand taste perception fully.

First, the exclusive focus on gastronomy students restricts the findings to a specific educational and disciplinary context, which may foreground sensory and technical interpretations of neurogastronomy while limiting insights into health-related, ethical, and broader societal perspectives that are more salient in other disciplinary or professional groups. Second, the use of Word Association Tests captures spontaneous cognitive representations but does not allow for deeper probing of participants’ reasoning or contextual explanations behind their associations. As such, certain dimensions may appear peripheral not because they are unimportant, but because they are less readily verbalized in rapid associative tasks. Additionally, participants’ varying degrees of implicit familiarity with the term neurogastronomy-although intentionally not controlled to avoid priming-may have influenced the structure of the observed semantic networks. These limitations suggest that future research would benefit from combining associative methods with qualitative interviews, cross-cultural comparisons, and diverse participant groups, thereby enabling a more nuanced and comprehensive exploration of how neurogastronomy is conceptualized across social and cultural contexts.

## Conclusion

The WAT method was used to investigate the cognitive associations of the concept of neurogastronomy amongst a large group of young Turkish gastronomy students. The lexical associations related to the perception of neurogastronomy and the hierarchical relationships among these associations have not been systematically analyzed previously. Neurogastronomy is a multidimensional term, one that brings together various phenomena such as taste, sensory perception, health, and scientific processes. The Turkish gastronomy students’ association of the term neurogastronomy with ‘science’, ‘brain’, and ‘sensory perception’ also reflects the broad scope and the interdisciplinary nature of the discipline.

The findings reported here highlight the hierarchy among the dimensions of neurogastronomy amongst students of gastronomy in Türkiye. The themes of ‘sensory experience’ and ‘taste perception’ were the most commonly mentioned and accounted for half of the study’s distribution of data. Themes that could be directly associated with neurogastronomy, such as health and creativity were, by contrast, mentioned only infrequently. This situation indicates that the understanding of neurogastronomy’s dimensions, as derived from the responses of Turkish gastronomy students, varies in terms of both depth and emphasis. These findings not only elucidate how a particular cohort of students views neurogastronomy but also establish a foundation for future cross-cultural studies to enhance and broaden its conceptual scope.

Beyond describing associative patterns, the present study contributes to the literature by offering one of the first systematic mappings of how the concept of neurogastronomy is cognitively structured among gastronomy students. By demonstrating how certain dimensions become cognitively foregrounded while others remain peripheral, the findings point to conceptual imbalances that may shape how future culinary professionals engage with neurogastronomy in practice. From an educational perspective, these results carry practical implications for gastronomy curricula. The relatively weak associations with health and creativity suggest that neurogastronomy is currently perceived primarily as a sensory-scientific concept rather than as a holistic framework integrating wellbeing, innovation, and interdisciplinary thinking. Integrating neurogastronomy more explicitly into gastronomy education-through sensory training, neuroscience-informed modules, and applied culinary experimentation-may help students develop a more balanced and comprehensive understanding of the field. In this respect, the study not only advances conceptual knowledge but also provides an empirical basis for curriculum development and pedagogical strategies aimed at strengthening the interdisciplinary integration of neuroscience and gastronomy. By identifying which dimensions of neurogastronomy are foregrounded or overlooked in students’ cognitive representations, the findings can inform educational interventions that better prepare future professionals to apply neurogastronomic principles in both academic and applied culinary contexts.

### Limitations and future directions

Notwithstanding the contributions of the present study, a number of limitations must be recognized. First, the findings are based exclusively on self-reported word association data collected from gastronomy students. Although both the WAT and FWA methods are established tools in research on cognitive representations and semantic networks, they depend on the participants’ immediate verbal outputs. Therefore, the results reflect perceived and consciously accessible associations rather than the neural or physiological processes underpinning flavour perception. While this approach is fitting for the exploratory and conceptual aims of the study, it may not fully capture the implicit or unconscious dimensions of neurogastronomic perception.

Second, the sample included only gastronomy students in Türkiye, which may restrict the generalization of results. While this group is very relevant because of their educational background and closeness to the field, their conceptualization of neurogastronomy might be different from that of professional chefs, food scientists, consumers, or people from different cultural backgrounds. In particular, perspectives emphasizing health, ethical, or societal aspects of food may be less pronounced in this sample. In addition, neurogastronomy is a rather new and interdisciplinary concept, and its cognitive representation can differ significantly across countries with different cuisines, educational systems, and scientific discourses.

Third, the methodological design was cross-sectional and descriptive in nature, mapping associative structures rather than causal or developmental processes. As such, the study does not allow one to draw conclusions on how conceptualizations of neurogastronomy may evolve over time or how educational exposure, professional experience, or sensory training may reshape these cognitive networks. Moreover, the methodological exclusion of words with low repetition frequencies-while methodologically justified-may have led to the omission of emerging or niche concepts that could become more prominent as the field develops.

These limitations may be addressed in various ways in future research. First, mixed-methods approaches that embed WAT/FWA in objective neuroscientific techniques, such as electroencephalography (EEG), eye-tracking, or facial expression analysis, may offer a more fine-grained view of how cognitive associations relate to actual sensory and neural responses. Such triangulation would raise the interpretative depth of neurogastronomy research by linking conceptual representation to embodied sensory processes. Another venue for future studies would be to use comparative and cross-cultural designs in order to investigate how gastronomy students, professional chefs, researchers, and consumers from different countries perceive neurogastronomy. This will help to test the cultural robustness of the identified dimensions, as well as to show how local culinary culture and educational framework shape the understanding of neurogastronomy.

Longitudinal research designs might also examine how conceptual associations with neurogastronomy evolve over the course of culinary education or professional practice. Research into the effects of focused sensory training, specific interdisciplinary course work, or specific exposure to neuroscientific gastronomic applications may yield a far greater understanding of how neurogastronomy might best be inculcated into the education and practice of gastronomy. These efforts would favor not only the theoretical development of this field but also its practical and pedagogic development.

## Data Availability

The raw data supporting the conclusions of this article will be made available by the authors, without undue reservation.

## Footnotes

1 E.g., “The cortical responses combine taste and smell to create the unified quality of flavor.” (Shepherd, 2012, p. 124).
