# Supplementary figures and images for: Exploring neurogastronomy: an analysis using the word association test and free word association
Source: Front Psychol. 2026 Feb 24;17:1771119. doi: 10.3389/fpsyg.2026.1771119 (PMC12971718; doi:10.3389/fpsyg.2026.1771119)

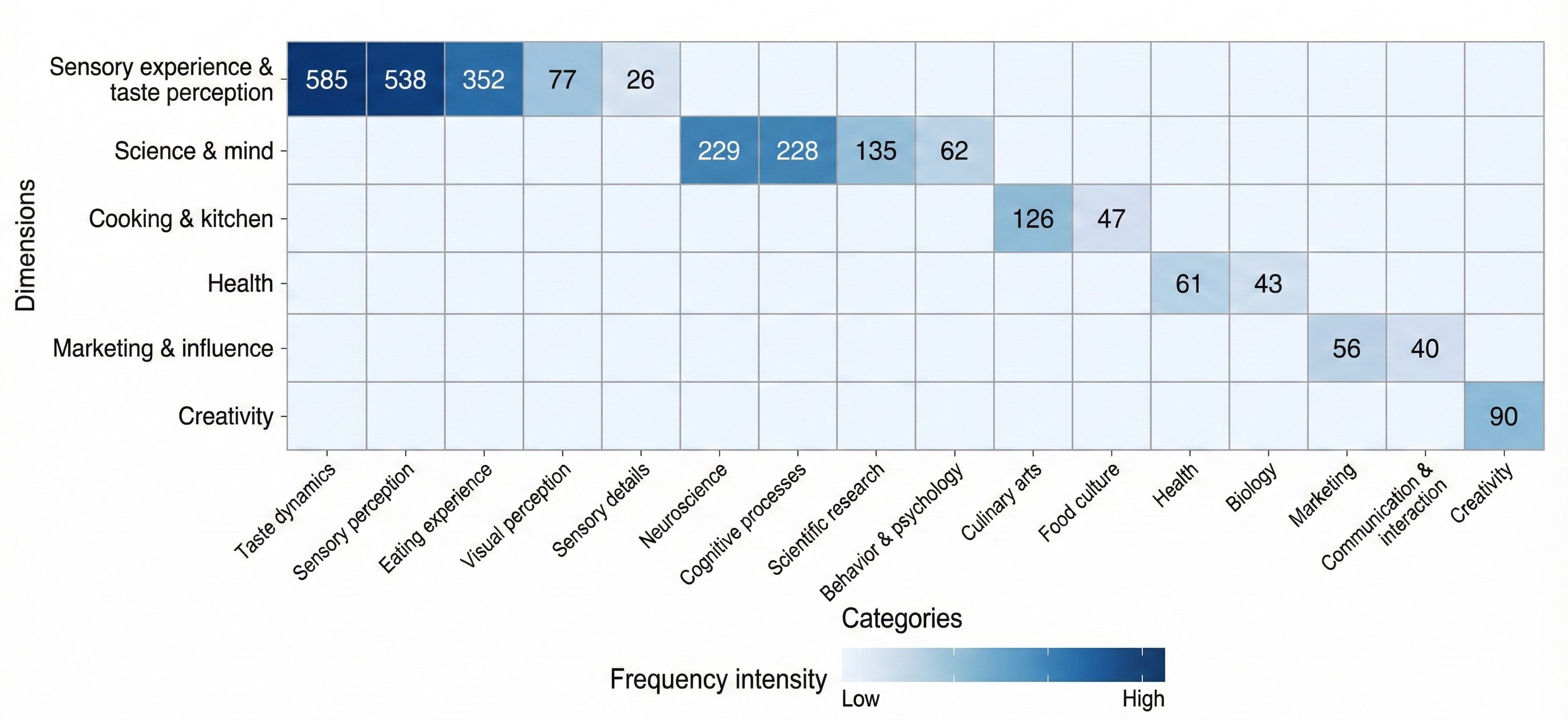

Supplement: Supplementary file 1 [file Supplementary_file_1.jpg]
